# Vascular architecture mapping reveals sex-specific changes in cerebral microvasculature with aging

**DOI:** 10.1162/imag_a_00066

**Published:** 2024-01-11

**Authors:** Anja Hohmann, Ke Zhang, Johann M.E. Jende, Christoph M. Mooshage, Kai Görgen, Lukas T. Rotkopf, Heinz-Peter Schlemmer, Philipp Vollmuth, Martin Bendszus, Wolfgang Wick, Felix T. Kurz

**Affiliations:** Department of Neurology, Heidelberg University Hospital, Heidelberg, Germany; Department of Diagnostic and Interventional Radiology, Heidelberg University Hospital, Heidelberg, Germany; Department of Neuroradiology, Heidelberg University Hospital, Heidelberg, Germany; Department of Psychiatry and Psychotherapy, Bernstein Center for Computational Neuroscience, Charité Berlin, Berlin, Germany; Department of Radiology, German Cancer Research Center, Heidelberg, Germany; Clinical Cooperation Unit Neurooncology, German Cancer Research Center, Heidelberg, Germany; Department of Neuroradiology, Geneva University Hospitals, Geneva, Switzerland

**Keywords:** Microvasculature, magnetic resonance imaging, cerebral vasculature, vascular architecture mapping, aging effects, sex differences

## Abstract

*Objectives:* Previous studies indicate region-specific age- and sex-related changes in cerebral microvasculature. Using whole-brain vascular architecture mapping (VAM), our objective was to map and assess these changes in human microvasculature *in vivo*.

*Materials and methods:* Cardiovascular healthy women (n = 40) and men (n = 32) with unifocal low-grade glioma, matched for age [range: 20-70 years] and BMI, were examined on the non-tumor hemisphere with a combined spin and gradient echo echo-planar imaging sequence at 3 T MRI. Vessel vortex curves were obtained by pair-wise plotting changes in relaxation rates R2* and R2 during contrast agent bolus passage, which each generate a set of VAM parameters that characterize microvascular properties, such as vessel type, lumen size, or blood flow. Averaged VAM values of cortical grey matter, white matter, putamen, globus pallidus, caudate nucleus, thalamus, insular cortex, and hippocampus were assessed for age- and sex-related changes.

*Results:* With age, dominant vessel types changed from capillaries to an arteriole-dominated profile, particularly in insula, thalamus, and globus pallidus. In white matter, blood flow velocity decreased significantly with aging for both sexes (r = −0.33, p = 0.004). In women, aging was associated with an increase in microvessel caliber, particularly in thalamus (r = 0.39, p = 0.01) and insula (r = 0.34, p = 0.03). In all grey matter areas, women had a higher microvessel density than men (4.33 ± 0.26ˑ10^2^ ms^-1/3^ vs. 4.18 ± 0.26ˑ10^2^ ms^-1/3^; p = 0.025, respectively).

*Conclusions:* Aging affects microvasculature differently across brain regions in women and men, especially in thalamus and insula.

## 1 Introduction

Morphology and function of cerebral microvasculature change with age, due to increasingly impaired endothelial function and altered angiogenesis, both of which are critical for repairing and adapting microvascular networks (Berthiaume et al., 2022; Brown & Thore, 2011; Gates et al., 2009; X. Xu et al., 2017). Histological studies show dilatation of capillaries and arterioles, increased vessel tortuosity, as well as a decrease in capillary density with age (Bell & Ball, 1981; Berthiaume et al., 2022; Lowerison et al., 2022; X. Xu et al., 2017). As arterioles and capillaries account for about 40% of cerebrovascular blood flow resistance (Iadecola, 2004), changes in microvascular morphology and arrangements significantly alter regional blood flow as well. Onset and extent of these changes are heterogenous across different brain regions and affected by both modifiable cardiovascular risk factors, such as elevated blood pressure, obesity or smoking, and non-modifiable risk factors, such as genetic predisposition and race (Murugesan et al., 2012; Sabbahi et al., 2021; Schager & Brown, 2020). Furthermore, human studies to date have not only shown sex differences in parameters such as vessel diameter, vascular tone, and basal blood flow, but also suggest that sex is an independent but interacting factor in age-related microvascular remodeling as well (Honarpisheh & McCullough, 2019; Huxley & Kemp, 2018; Lohner et al., 2022; Pabbidi et al., 2018).

A recently introduced non-invasive approach to characterize alterations in cerebral microvessels is vessel architecture imaging or vascular architecture mapping (VAM) (Emblem et al., 2013; Stadlbauer, Zimmermann, Kitzwögerer, et al., 2017; K. Zhang et al., 2019). VAM is a specialized MRI method that has shown potential as an imaging biomarker for microvascular alterations and therapy effects in brain tumor patients (Emblem et al., 2013; Stadlbauer et al., 2019), but also vascular changes in patients with dementia, (Choi et al., 2020) or cerebrovascular insults (C. Xu et al., 2011).

The basic principle underlying VAM are different sensitivities of gradient echo (GE) and spin echo (SE) relaxation rates, R_2_* and R_2,_ respectively, to vascular arrangements, during contrast agent bolus administration (Emblem et al., 2013; K. Zhang et al., 2019). While R_2_ is mainly sensitive to small caliber vessels with diameters below 10 µm, R_2_* is more sensitive to larger caliber vessels (Emblem et al., 2013; Kiselev et al., 2005; Kurz et al., 2016, 2017). The combined examination of dynamic changes in relaxation rates for both R_2_* and R_2_ during contrast bolus passage therefore provides information about vascular architecture properties within the imaging voxel: plotting R_2_* against R_2_ for every sampled time-point results in time-parametrized vortex curves, or vascular hysteresis loops (VHL), whose shape, slope, and direction are characteristic for the vascular arrangement (Emblem et al., 2013; Stadlbauer, Zimmermann, Heinz, et al., 2017).

Preliminary studies have shown the applicability of VAM in neurooncology; however, no study has examined the effects of physiological age-related morphological changes on VAM parameters in healthy tissue yet. Furthermore, it remains unclear how and to what extent sex influences changes in microvascular arrangements, as measured by VAM. Yet, these effects certainly need to be taken into account, for example, when quantifying therapy responses in neurooncology with VAM (Stadlbauer et al., 2019; Stadlbauer, Zimmermann, Oberndorfer, et al., 2017). While there is a lack of studies examining regional cerebral microvasculature between men and women, neuroimaging studies have repeatedly shown neuroanatomical differences, in, for example, regional volume and tissue density, local connectivity or sex hormone receptor density, in brain regions such as thalamus, putamen, hippocampus, and insular cortex (Gong et al., 2009; Liu et al., 2020; Ruigrok et al., 2014).

This study aims to test the hypothesis that aging has a different impact on VAM parameters of healthy brain tissue in different regions and that the extent of vascular changes varies between women and men.

Region-specific analyses of VAM focused on anatomical areas that we hypothesize to be particularly sensitive to sex-specific changes with age, either regions with known macrostructural changes, such as aging-related atrophy, or regions attributed to cognitive functions that decline with normal aging, like memory or information processing, e.g., thalamus, hippocampus, and insular cortex (Cherubini et al., 2009; Fama & Sullivan, 2015; Gogolla, 2017; Nobis et al., 2019).

## 2 Materials and Methods

Subjects were chosen from an institutional imaging data base of n = 117 patients with clinically and radiologically stable unifocal low grade brain tumors (WHO grade 1-2), and no history of tumor specific therapy other than surgical resection, i.e., no previous or ongoing radio- or chemotherapy. Participants with any diagnosis of cerebrovascular or cardiovascular disease, arterial hypertension, hypercholesterinemia or diabetes mellitus as well as smokers were excluded. Subjects with lesions showing midline shift or midline infiltration as well as scans with imaging artefacts affecting the contralateral healthy hemisphere were excluded. In total, 72 subjects (42 female, 30 male) between 20 and 70 years of age were included in the analysis, for detailed demographics see Table 1. Only data from the healthy contralateral non-tumor hemispheres and only one scan per subject were included in statistical analysis.

**Table 1.** Demographics of the cohort (n = 72, 42 females).

Variable	Cohort (mean)
Sex (% female)	58.3
Age (years)	37.6 ± 12.0 [20-70]
-Women	37.7 ± 11.9
-Men	37.3 ± 12.4
BMI (kg/m^2^)	25.4 ± 3.9 [18.4-35.5]
-Women	24.8 ± 4.2
-Men	26.3 ± 3.2
KPS	96.3 ± 6.2 [80-100]
-Women	96.9 ± 5.2
-Men	95.3 ± 7.3

Mean values ± SD, [range].

KPS: Karnofsky performance status. BMI: body mass index.

All subjects were examined between 02/2017 and 12/2019 as part of their regular clinical follow-up on a 3 T Prisma Siemens scanner (Siemens Healthineers AG, Erlangen, Germany) in the Department of Neuroradiology at Heidelberg University Hospital. The local ethics committee of Heidelberg University approved the data evaluation and the requirement for informed consent was waived.

### 2.1 MR image acquisition

The standardized protocol included pre- and postcontrast T1-weighted imaging, T2-weighted imaging, fluid-attenuated inversion recovery (FLAIR), diffusion- and susceptibility-weighted imaging, as well as VAM. We obtained whole-brain high-resolution VAM by using multiband accelerated spin and gradient echo (SAGE) echo planar imaging (EPI). The readout-slices for whole-brain coverage were doubled by applying multiband excitation and blipped-CAIPI (controlled aliasing in parallel imaging) techniques, which allowed tracking MR signal changes in GE and SE contrasts simultaneously, for detailed description see also K. Zhang et al. (2019). A total brain coverage of 13 cm, with 24 slices each for dual GE/SE readout, was obtained after application of contrast agent (0.1 mmol/kg-bodyweight gadoterate meglumine (Dotarem, Guerbet, France)) at a rate of 4 ml/s. Total image acquisition time was 1.5 minutes, corresponding to 15 seconds baseline and 75 seconds for contrast agent administration and post-contrast image acquisition.

High-resolution anatomical T1-weighted images were obtained with a three-dimensional magnetization-prepared rapid gradient echo (MP RAGE) sequence.

Sequence parameters were as follows: MR SAGE-EPI: TR = 1500 ms, TE = 22 ms (GE) and 90 ms (SE), matrix dimensions = 120 x 120, slice thickness = 4,5 mm, interslice gap = 0.9 mm, voxel size = 2 x 2 x 5.4 mm^3^, multiband factor = 2, parallel imaging factor = 3, acquisition time 1.5 minutes. MP RAGE: TR = 1790 ms, TE = 3.7 ms, flip angle (FA) = 15°, slice thickness = 1 mm, field of view (FOV) = 250 × 250, and voxel size = 0.78 x 0.78 x 1 mm^3^. Dynamic susceptibility contrast (DSC) MR perfusion: TR = 2220 ms, TE = 37 ms, FA = 90°, FOV = 240 × 240, during contrast bolus administration.

### 2.2 Data postprocessing

**Fig. 1.**
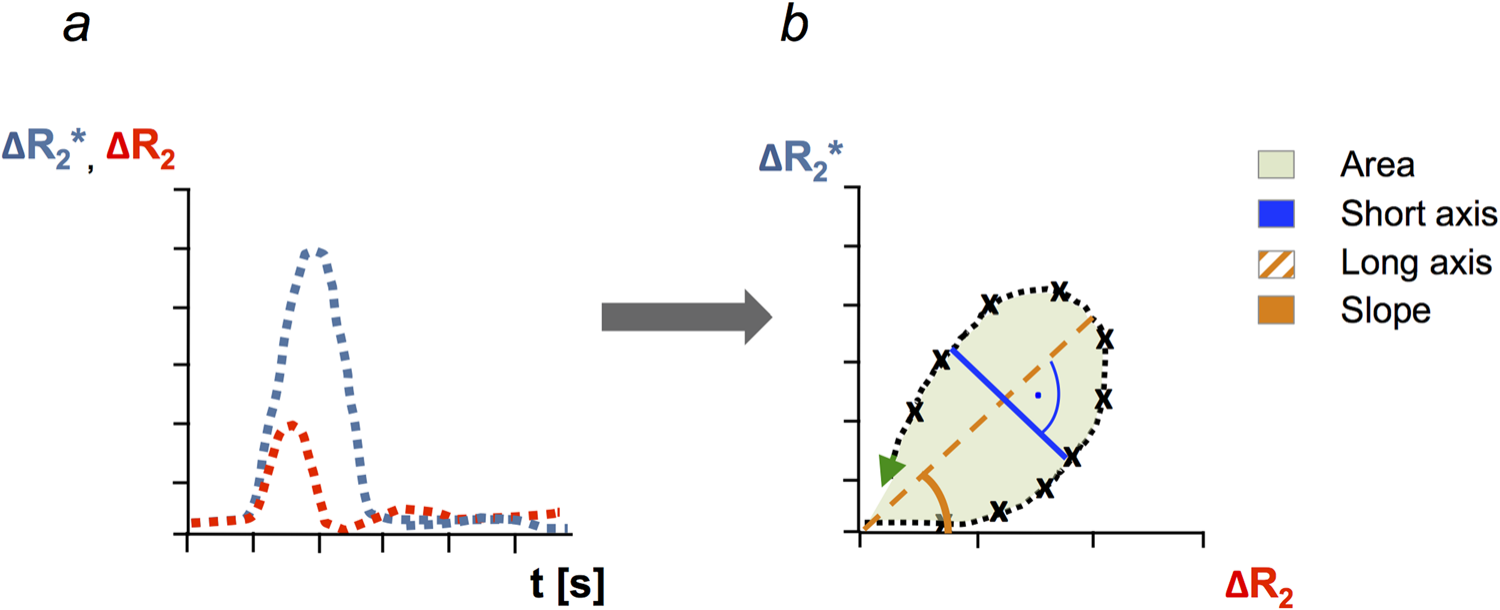
Calculating the vessel vortex curve and selected parameters. (a) Changes in spin-echo (SE) and gradient-echo (GE) relaxation rates (ΔR_2,_ ΔR_2_*, respectively) over time, relative to baseline. In this case, the SE signal peaks before the GE signal. (b) Pairwise plotting of ΔR_2,_ versus ΔR_2_* yields the vessel vortex curve, in this case, in counterclockwise direction (green arrow). See also detailed description in the main text.

VAM analysis was conducted using custom-built MATLAB code (MathWorks, Natick, MA) after motion correction and alignment in SPM12 (Wellcome Center for Human Neuroimaging, UCL London, UK). SE and GE relaxation rates were calculated from average baseline signal intensity, S_0_, and observed signal intensities during contrast bolus administration, S(t), with $\Delta \;R\left(t\right)=-\frac{1}{TE}\ln\left(\frac{S\left(t\right)}{S_{0}}\right)$. After correction for contrast agent leakage (Boxerman et al., 2006), relaxation rate curves were fitted to a gamma-variate function (Rausch et al., 2000) and then plotted pairwise against each other over time, generating a time-parametrized vascular hysteresis loop or vessel vortex curve (Emblem et al., 2013; K. Zhang et al., 2019). Six VAM parameters were calculated from the VHL, as described in detail previously (K. Zhang et al., 2019); see also Figure 1.

Distance map I was defined as the maximum distance between ascending and descending branch of the vortex curve, while the area of the curve was termed vessel type indicator (VTI). Values of both I and VTI were signed according to the direction of the vortex curve (positive, if the loop transversed clockwise, and negative, if it transversed counter-clockwise). The slope of the long axis of the vortex curve was termed caliber gradient indicator (CGI), since higher values have been shown to indicate both larger vessel calibers and higher amounts of deoxygenated blood, as in venules (Emblem et al., 2013), while the length of the long axis has been shown to correlate with cerebral blood volume fraction (K. Zhang et al., 2019), and was therefore abbreviated BVF. The length of the short axis, that is perpendicular to the long axis, has been shown to identify local minima of both vessel caliber and differences in oxygen saturation (Emblem et al., 2013), and was therefore termed capillary bed identifier (CBI). Vascular-induced bolus peak-time shift (VIPS) was calculated from the temporal shift between the time-to-peak of SE and GE-EPI signal curves, with negative values indicating a relative predominance of slow-flowing venules and capillaries (Stadlbauer, Zimmermann, Heinz, et al., 2017).

We calculated vessel size index (VSI) and microvessel density Q according to C. Xu et al. (2013). Calculation of relative cerebral blood volume (rCBV) was based on the DSC-perfusion sequence as in Buschle et al. (2018).

Finally, whole-brain parameter maps were generated for every parameter and scan, yielding 9 parameter maps for each of the 72 scans. We coregistered subjects’ anatomical T1-weighted images to their functional VAM scans, and spatially normalized to standardized space (Montreal Neurological Institute, MNI) in SPM12 using mutual information affine registration with tissue probability maps and 4^th^ degree B-spline interpolation (D’Agostino et al., 2003). With these individual transformation parameters, all VAM maps and tissue segmentations per subject were subsequently spatially normalized. Hereinafter, each non-healthy hemisphere was set to zero and individual half-brain parameter maps were averaged separately per hemisphere. For visualization purposes only, averaged hemisphere maps were then joined together.

### 2.3 Volume of interest analysis

**Fig. 2.**
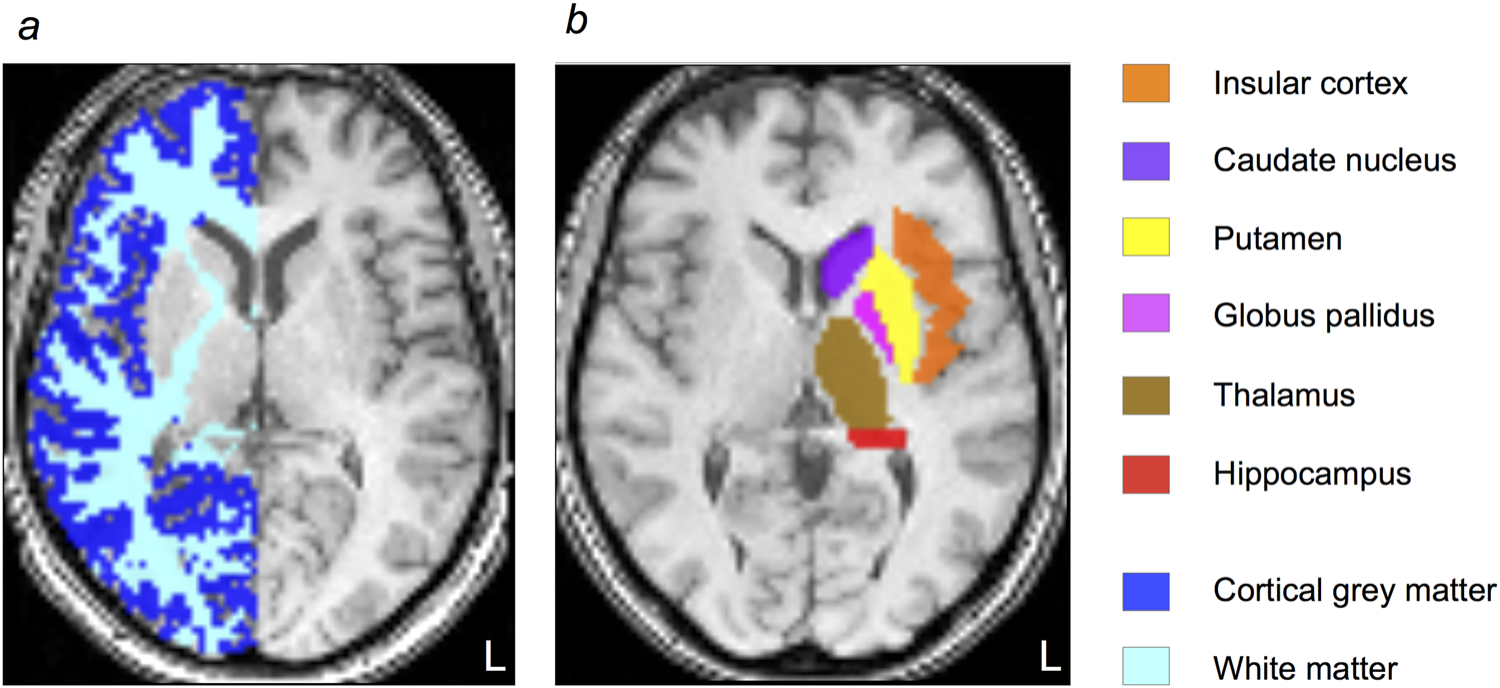
Analyzed volumes of interest. (a) Automatically segmented individual grey and white matter VOIs on a spatially normalized axial T1-weighted image of a subject with left hemispheric tumor (male, 39 years old). (b) Anatomical volumes of interest (VOIs) on a spatially normalized axial T1-weighted image of a subject with right hemispheric tumor (female, 39 years old). For each subject, only the hemisphere contralateral to the lesion was analyzed, generating mean values for a total of 8 VOIs per subject.

Individual volumes of interests (VOIs) for cortical grey matter (cGM; whole-hemisphere supratentorial cortical grey matter) and supratentorial white matter (WM) were generated for each subject with automated tissue segmentation in FSL FAST (Y. Zhang et al., 2001) (FMRIB, Oxford, UK) on coregistered, brain-extracted anatomical T1-images (see Fig. 2a). Both infratentorial region (brainstem and cerebellum) and subcortical grey matter (basal ganglia, thalamus) were excluded from cGM- and WM-masks using the automated anatomical labeling atlas 3 (AAL3) (Rolls et al., 2020; Tzourio-Mazoyer et al., 2002) with 2 x 2 x 2 mm^3^ voxel size. Additionally, we identified the left and right insular cortex, thalamus, putamen, globus pallidus (GP), caudate nucleus (CN), and hippocampus using the AAL3 (see Fig. 2b).

Visual inspection and correction, if necessary, of VOIs was performed by a neurologist (AH) with 6 years of experience in neuroimaging and a trained neuroradiologist (FTK) with 10 years of clinical experience.

Only data from the healthy contralateral hemisphere were included in further analysis, that is, for example, data from left hemispheric VOIs for subjects with right-sided lesions. In summary, 8 VOIs per subject were analyzed.

### 2.4 Statistical analysis

Mean values with 95% confidence interval and standard deviations were calculated for each VOI and VAM parameter per subject. Statistical data analysis was performed in MATLAB 2019a and GraphPad Prism 9 (GraphPad Software, Inc., Boston, MA, USA). Outliers were analytically identified with the ROUT method (Q = 1%). We tested for Gaussian normal distribution with the D'Agostino-Pearson omnibus normality test. In case of a Gaussian normal distribution, we used t-tests for comparisons of two groups and Pearson correlation coefficients for correlation analysis. If data were not Gaussian distributed, we used the Mann–Whitney test for comparisons of two groups, and nonparametric Spearman’s Rho for correlation analysis. Partial correlations were used to assess the relationship between age and VAM parameter mean value for each VOI, while controlling for BMI. A p-value <0.05 was considered statistically significant for all tests. We adjusted for multiple comparisons between groups by controlling the false discovery rate (FDR) using the original method by Benjamini and Hochberg (Q = 5%) (Benjamini & Hochberg, 1995; Genovese et al., 2002).

## 3 Results

**Fig. 3.**
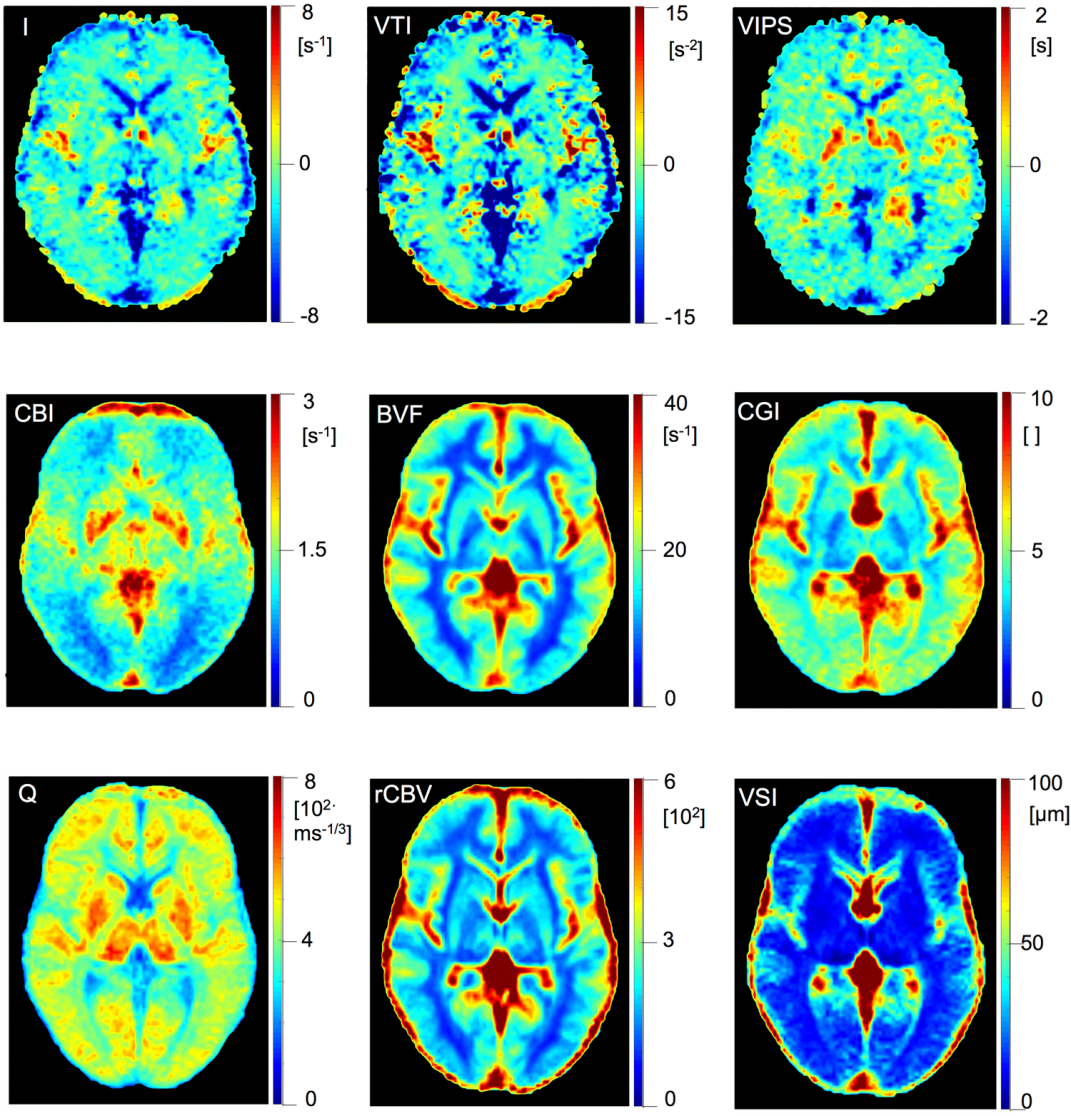
Axial representations of averaged VAM parameter maps. Individual half-brain maps were spatially normalized and averaged across healthy hemispheres (n = 36 right, n = 36 left). Averaged hemisphere maps were then joined together for visualization purposes only. Slice coordinates (Z-axis) in MNI standard space: z = 4. For detailed information on parameters, see main text. [VTI: vessel type indicator, VIPS: vascular-induced bolus peak-time shift, CBI: capillary bed identifier, BVF: cerebral blood volume fraction, GCI: caliber gradient indicator, rCBV: relative cerebral blood volume, VSI: vessel size index].

In total, 72 subjects (42 female, 30 male) were included in the analysis, with 36 right and 36 left hemispheres. Axial representation of averaged whole-brain maps (n = 72) can be seen in Figure 3. As hemisphere groups did not differ for age, BMI, or number of females, subsequent analyses of VOIs was performed across hemispheres. We obtained 9 averaged VAM parameters per VOI (8 VOIs) and patients, resulting in 5184 averaged VAM parameters. Based on the ROUT-method, a total of 15 of those parameter values were identified as extreme outliers and excluded from further analysis.

Women and men did not differ significantly for age (37.7 ± 11.9 years vs. 37.3 ± 12.4 years, p = 0.88) and BMI (24.8 ± 4.2 kg/m^2^ vs. 26.3 ± 3.2 kg/m^2^, p = 0.10). Furthermore, there was no correlation between age and BMI across the cohort (r = 0.19, p = 0.12). However, when analyzing both sexes separately, there was an increase in BMI with age for male subjects (r = 0.52, p = 0.004), an association not observed in female subjects in our cohort (r = 0.02, p = 0.91). Consequently, further correlations with age were adjusted for BMI.

### 3.1 Age-related region-specific changes in vascular architecture

#### 3.1.1 Cortical grey matter

When analyzing individual whole-hemisphere cortical grey matter (cGM) VOIs, we found no significant correlation between age and any of the analyzed vascular architecture parameters; see Table 2.

**Table 2.** Partial correlations for VAM parameter mean value with age, controlled for BMI, for selected brain regions, for all subjects (n = 72).

Region		I	VTI	VIPS	CGI	CBI	BVF	Q	VSI	rCBV
cGM	r	0.17	0.14	-0.03	0.05	-0.10	0.01	-0.01	-0.11	-0.08
	p	*0.159^P^*	*0.246^P^*	*0.822^P^*	*0.702^P^*	*0.420^P^*	*0.942^P^*	*0.941^P^*	*0.380^P^*	*0.498^P^*
WM	r	-0.12	0.01	**-0.33**	0.20	-0.13	0.13	0.09	0.04	**0.36**
	p	*0.330^S^*	*0.953^S^*	***0.004^P^***	*0.101^P^*	*0.272^P^*	*0.289^P^*	*0.468^P^*	*0.753^S^*	***0.002^S^***
Caudate nucleus	r	0.00	0.03	0.07	**0.37**	-0.14	0.04	**-0.38**	**0.31**	-0.03
	p	*0.996^P^*	*0.794^P^*	*0.572^S^*	***0.002^P^***	*0.257^P^*	*0.750^P^*	***0.001^P^***	***0.009^P^***	*0.830^P^*
Putamen	r	0.15	0.18	0.01	0.13	0.02	0.13	0.11	0.06	**0.26**
	p	*0.207^S^*	*0.132^P^*	*0.907^P^*	*0.265^P^*	*0.873^S^*	*0.296^P^*	*0.346^P^*	*0.640^P^*	***0.031^P^***
Globus pallidus	r	**0.27**	**0.34**	0.05	0.10	0.04	0.18	0.11	0.22	**0.35**
	p	***0.023^S^***	***0.004^S^***	*0.675^P^*	*0.391^P^*	*0.767^P^*	*0.137^S^*	*0.362^P^*	*0.069^P^*	***0.003^P^***
Thalamus	r	0.23	**0.24**	0.16	**0.38**	-0.09	**0.24**	-0.11	**0.35**	**0.37**
	p	*0.055^P^*	***0.048^S^***	*0.181^S^*	***0.001^P^***	*0.458^P^*	***0.045^P^***	*0.351^P^*	***0.003^S^***	***0.002^S^***
Insula	r	**0.32**	**0.30**	**0.28**	**0.25**	0.02	0.13	-0.17	**0.27**	**0.38**
	p	***0.007^P^***	***0.010^S^***	***0.017^S^***	***0.033^P^***	*0.860^S^*	*0.279^P^*	*0.152^P^*	***0.022^S^***	***0.001^P^***
Hippocampus	r	0.03	-0.01	-0.10	0.20	-0.02	0.05	0.05	0.03	0.21
	p	*0.794^P^*	*0.933^S^*	*0.413^S^*	*0.089^P^*	*0.841^S^*	*0.667^S^*	*0.656^P^*	*0.800^S^*	*0.076^P^*

Bold r-values and bold italic p-values indicate significant correlations (p < 0.05.)

^P^p-Value obtained from Pearson correlation analysis.

^S^p-Value obtained from Spearman correlation analysis.

r: correlation coefficient, p: p-value, cGM: cortical grey matter, WM: white matter, VTI: vessel type indicator, VIPS: vascular-induced bolus peak-time shift, GCI: caliber gradient indicator, CBI: capillary bed identifier, BVF: cerebral blood volume fraction, VSI: vessel size index, rCBV: relative cerebral blood volume.

**Fig. 4.**
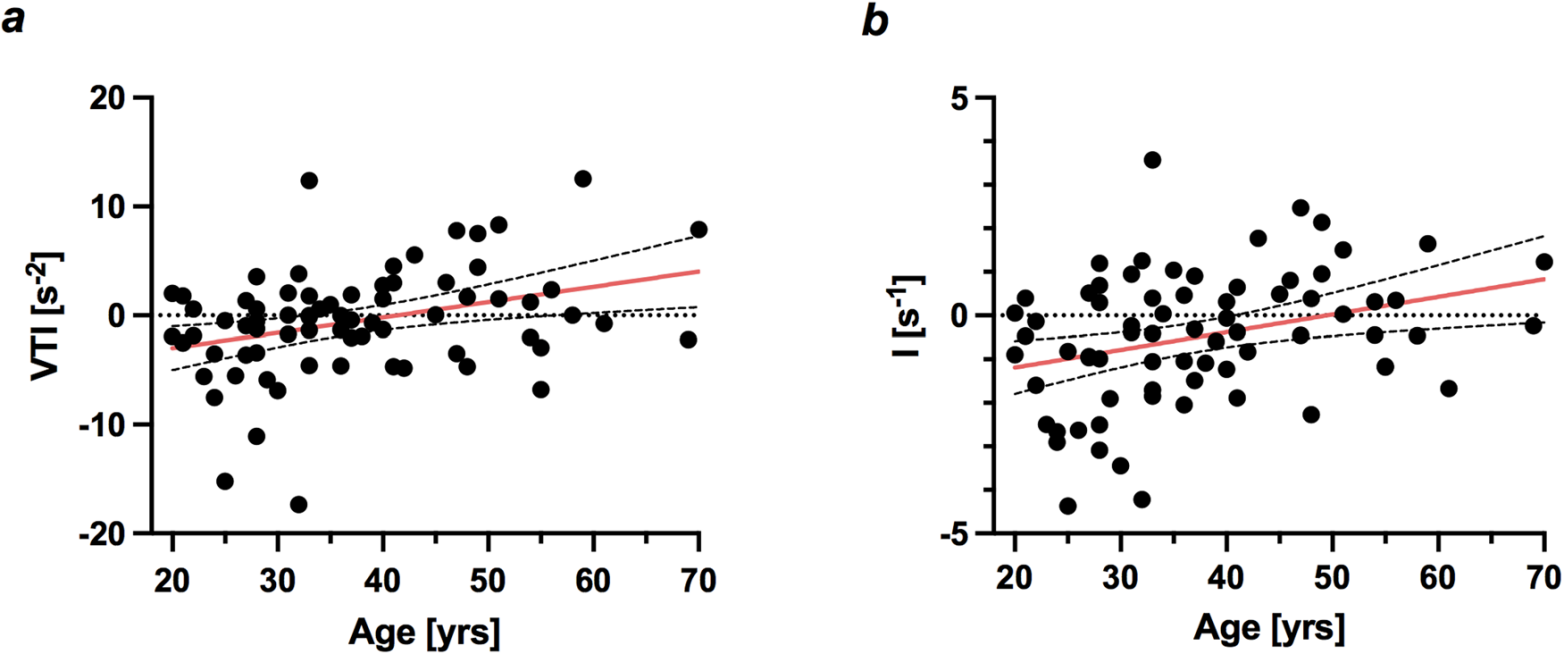
Age-related changes in (a) vessel type indicator (VTI) and (b) parameter I in insular cortex. N = 72. Red line indicates line of best fit for simple linear regression, with 95% confidence intervals (dashed lines).

However, focusing on the insular cortex, a highly specialized cortical subregion, we found an age-related increase in vessel-caliber sensitive parameters VSI and CGI as well as a significant increase in relative cerebral blood volume rCBV with age; see Table 2. Furthermore, all analyzed brain regions exhibited negative values for distance map I and vessel type indicator VTI, pointing towards a relative dominance of venules and capillary-type vessels in healthy tissue. In insular cortex, age was positively correlated with both I and VTI when controlling for BMI, that is, older age was associated with less negative or even positive values; see Figure 4a and 4b and Table 2. Furthermore, age was positively correlated with VIPS (r = 0.28, p = 0.02).

In hippocampus, no significant correlation was found between VAM parameters and age.

#### 3.1.2 Basal ganglia and thalamus

**Fig. 5.**
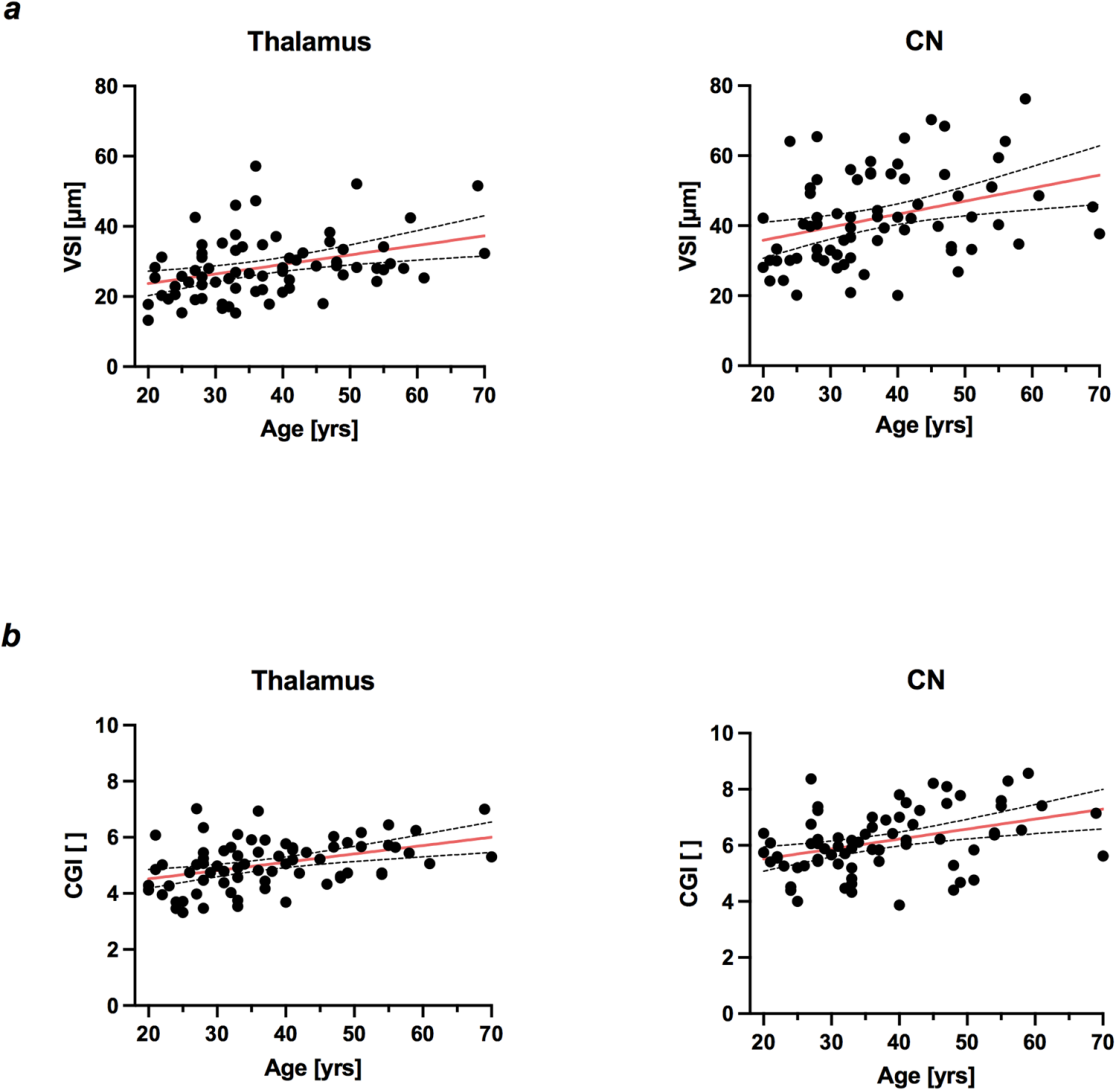
Age-related changes in (a) vessel size index (VSI) and (b) caliber gradient indicator (CGI). Mean values for thalamus and caudate nucleus (CN). N = 72. Red line indicates line of best fit for simple linear regression, with 95% confidence intervals (dashed lines).

A variety of age-related changes in microvasculature were observed in thalamus: both vessel-caliber sensitive parameters CGI and VSI as well as vessel type indicator VTI increased with age; see Table 2 and Figure 5a and 5b. Additionally, both rCBV (r = 0.37, p = 0.002) and blood volume fraction BVF (r = 0.23, p = 0.045) increased significantly with age, while no age-related changes in BVF were observed for the other analyzed brain areas. CGI and VSI increased in caudate nucleus (CN) with age as well; see Figure 5a and 5b and Table 2. Older age (r = −0.38, p = 0.001) was associated with a decrease in microvessel density Q in CN.

With age, rCBV increased in both globus pallidus (GP) (r = 0.35, p = 0.003) and putamen (r = 0.26, p = 0.03). No other significant age-related changes were measured in putamen. In GP, older age was associated with higher mean values for both I and VTI; see Table 2.

#### 3.1.3 White matter

**Fig. 6.**
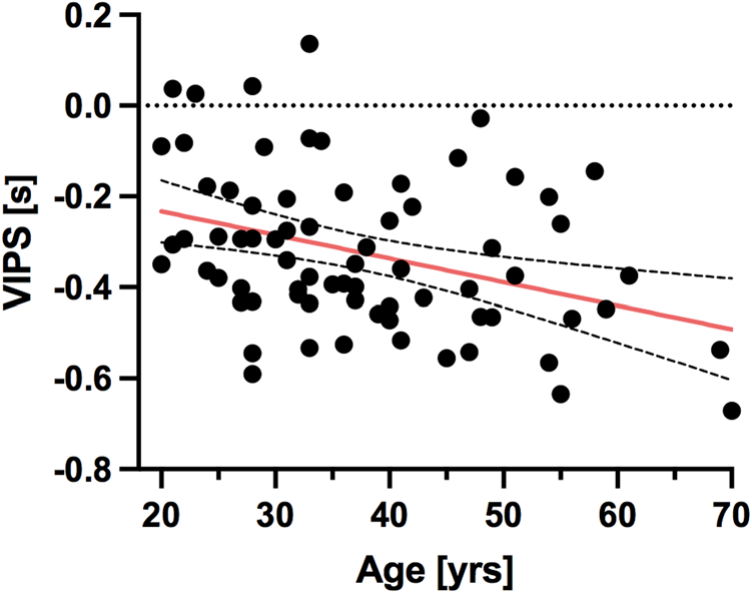
Age-related decreases in white matter for parameter VIPS, a measure for blood flow velocity (Mean values, n = 72). Red line indicates line of best fit for simple linear regression, with 95% confidence intervals (dashed lines).

VIPS, an indicator for blood flow velocity, decreased significantly with age in white matter (r = −0.33, p = 0.004); see Figure 6. rCBV increased with age (r = 0.36, p = 0.002). No other changes with age were observed for WM.

### 3.2 Sex-specific differences in microvascular architecture

#### 3.2.1 Cortical grey matter

**Fig. 7.**
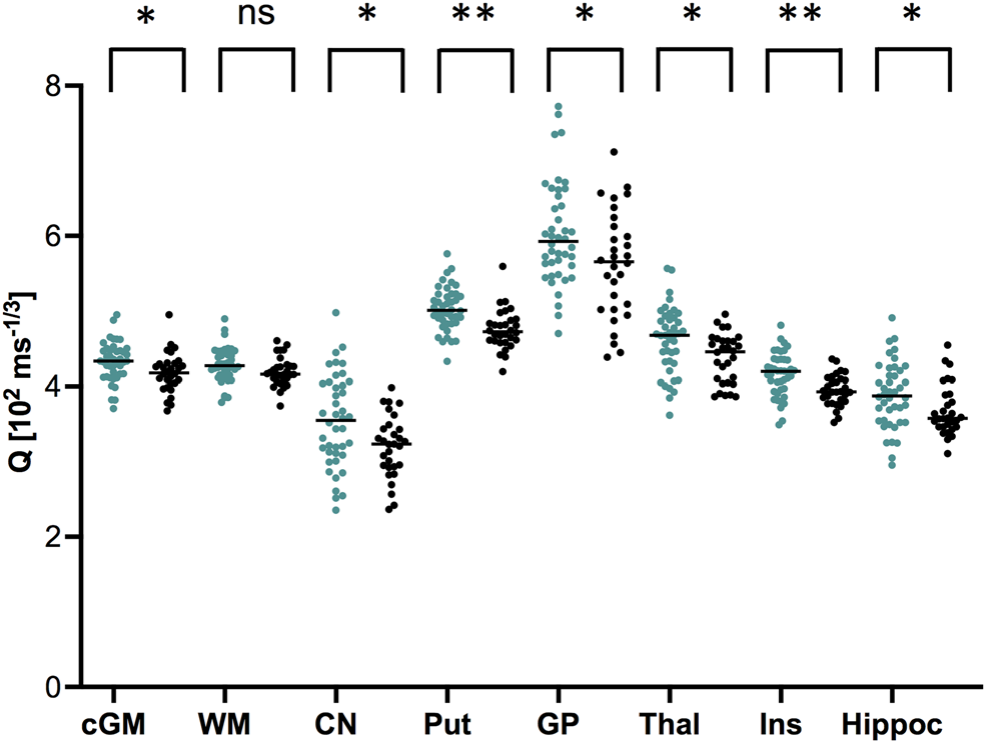
Sex-specific differences in microvessel density (Q) for different brain regions. Mean values per VOI, for women (dark green, n = 42) and men (black, n = 30). Line indicates median value. Asterisks indicate FDR-adjusted p-values for unpaired t-tests between women (n = 42) and men (n = 30). *p < 0.05,**p < 0.01. ns: not significant. [cGM: cortical grey matter, WM: white matter, CN: caudate nucleus, Put: putamen, GP: globus pallidus, Thal: thalamus, Ins: insular cortex, Hippoc: hippocampus].

Mean values for age-matched female (n = 42) and male (n = 30) subjects are displayed in Table 3. On average, women had higher microvessel density Q in all analyzed cortical grey matter VOIs, that is, whole-hemisphere cGM (p = 0.025), insular cortex (p = 0.004), and hippocampus (p = 0.045), but not in WM (p = 0.09) (see Fig. 7).

**Table 3.** Sex-specific mean values for VAM parameters in selected brain regions, for women (n = 42) and men (n = 30).

Region		I	VTI	VIPS	CGI	CBI	BVF	VSI	Q	rCBV
[unit]		[s^-1^]	[s^-2^]	[s]	[ ]	[s^-1^]	[s^-1^]	[µm]	[10^2^ˑms^-1/3^]	[10^2^]
cGM	Women	-2.52 ± 0.90	-6.04 ± 2.71	-0.43 ± 0.17	5.72 ± 0.50	1.48 ± 0.29	20.84 ± 4.04	36.22 ± 3.59	4.33 ± 0.26	3.02 ± 0.09
	Men	-1.91 ± 0.78	-4.34 ± 2.25	-0.37 ± 0.17	5.77 ± 0.56	1.35 ± 0.23	18.98 ± 3.68	37.44 ± 3.7	4.18 ± 0.26	3.02 ± 0.12
	p*-value*	***0.032^m^***	***0.032^m^***	*0.596^t^*	*0.695^t^*	***0.027^m^***	*0.122^t^*	*0.193^t^*	***0.025^t^***	*0.878^t^*
WM	Women	-1.44 ± 0.54	-2.82 ± 1.35	-0.32 ± 0.18	4.23 ± 0.50	1.18 ± 0.20	11.23 ± 2.22	18.78 ± 1.49	4.28 ± 0.23	1.58 ± 0.09
	Men	-1.15 ± 0.46	-2.14 ± 1.03	-0.32 ± 0.16	4.09 ± 0.58	1.12 ± 0.17	10.16 ± 1.94	19.09 ± 1.97	4.20 ± 0.19	1.60 ± 0.12
	p*-value*	*0.084^m^*	*0.076^m^*	*0.982^t^*	*0.566^t^*	*0.093^m^*	*0.122^t^*	*0.438^t^*	*0.086^t^*	*0.878^m^*
Caudate	Women	-2.60 ± 1.96	-6.25 ± 4.15	-0.41 ± 0.50	6.01 ± 1.05	1.43 ± 0.31	16.78 ± 3.79	40.15 ± 12.72	3.54 ± 0.61	2.42 ± 0.32
nucleus	Men	-2.22 ± 1.43	-3.98 ± 2.73	-0.45 ± 0.33	6.31 ± 1.19	1.21 ± 0.22	15.46 ± 3.39	45.48 ± 12.94	3.20 ± 0.41	2.53 ± 0.32
	p*-value*	*0.605^m^*	*0.141^m^*	*0.873^t^*	*0.566^t^*	***0.016^t^***	*0.177^t^*	*0.138^t^*	***0.025^t^***	*0.434^m^*
Putamen	Women	-2.10 ± 1.30	-3.77 ± 2.78	-0.25 ± 0.27	4.16 ± 0.67	1.67 ± 0.35	16.08 ± 4.40	12.3 ± 1.85	5.03 ± 0.29	1.93 ± 0.27
	Men	-1.70 ± 0.82	-2.99 ± 2.29	-0.27 ± 0.24	4.08 ± 0.64	1.55 ± 0.25	14.12 ± 2.86	13.54 ± 1.64	4.75 ± 0.27	2.01 ± 0.21
	p*-value*	*0.224^t^*	*0.283^m^*	*0.873^t^*	*0.695^t^*	*0.093^m^*	*0.122^t^*	***0.024^t^***	***0.004^m^***	*0.428^t^*
Globus	Women	-1.33 ± 1.68	-3.63 ± 5.67	0.34 ± 0.67	3.20 ± 0.75	2.51 ± 0.51	16.02 ± 4.21	10.36 ± 3.47	6.03 ± 0.69	1.77 ± 0.29
pallidus	Men	-1.01 ± 0.86	-2.94 ± 3.65	0.66 ± 0.73	2.96 ± 0.54	2.42 ± 0.46	14.44 ± 2.29	11.68 ± 3.6	5.62 ± 0.71	1.86 ± 0.26
	p*-value*	*0.605^m^*	*0.704^m^*	*0.464^t^*	*0.566^t^*	*0.557^t^*	*0.122^t^*	*0.159^t^*	***0.025^t^***	*0.428^t^*
Thalamus	Women	-1.34 ± 1.61	-3.45 ± 3.52	-0.03 ± 0.35	4.99 ± 0.78	1.73 ± 0.32	18.27 ± 4.14	26.66 ± 7.00	4.61 ± 0.44	2.63 ± 0.35
	Men	-0.78 ± 1.24	-2.01 ± 3.30	-0.06 ± 0.29	5.11 ± 1.00	1.69 ± 0.31	17.28 ± 3.49	31.00 ± 10.75	4.37 ± 0.33	2.86 ± 0.56
	p*-value*	*0.224^t^*	*0.186^m^*	*0.873^m^*	*0.695^t^*	*0.621^m^*	*0.333^t^*	*0.084^t^*	***0.025^t^***	*0.132^t^*
Insula	Women	-0.70 ± 1.59	-0.94 ± 5.49	-0.06 ± 0.19	6.32 ± 0.70	1.55 ± 0.33	24.95 ± 5.58	32.28 ± 7.40	4.16 ± 0.29	3.22 ± 0.49
	Men	-0.17 ± 1.39	0.03 ± 4.40	0.00 ± 0.22	6.39 ± 0.77	1.39 ± 0.27	22.9 ± 5.26	36.78 ± 7.47	3.95 ± 0.21	3.31 ± 0.47
	p*-value*	*0.224^m^*	*0.283^m^*	*0.684^m^*	*0.695^t^*	***0.027^m^***	*0.122**^m^***	***0.024^m^***	***0.004^t^***	*0.551^t^*
Hippo-	Women	-2.49 ± 1.59	-6.23 ± 4.45	-0.47 ± 0.44	5.95 ± 0.79	1.64 ± 0.32	21.87 ± 4.63	42.12 ± 8.92	3.88 ± 0.44	3.97 ± 0.63
campus	Men	-2.30 ± 1.34	-6.30 ± 4.40	-0.62 ± 0.42	6.19 ± 0.81	1.63 ± 0.26	21.17 ± 3.37	48.71 ± 11.48	3.68 ± 0.34	4.34 ± 0.76
	p*-value*	*0.605^t^*	*0.704^m^*	*0.637^m^*	*0.566^t^*	*0.621^m^*	*0.485^t^*	***0.024^t^***	***0.045^t^***	*0.132^t^*

p-Values (in italic) are adjusted for multiple comparisons using the FDR approach (Q=5%). Bold italic values indicate significant p-values.

^t^p-Value obtained from unpaired t-test.

^m^p-value obtained from Mann-Whitney test.

cGM: cortical grey matter, WM: white matter, VTI: vessel type indicator, VIPS: vascular-induced bolus peak-time shift, GCI: caliber gradient indicator, CBI: capillary bed identifier, BVF: cerebral blood volume fraction, VSI: vessel size index, rCBV: relative cerebral blood volume.

**Fig. 8.**
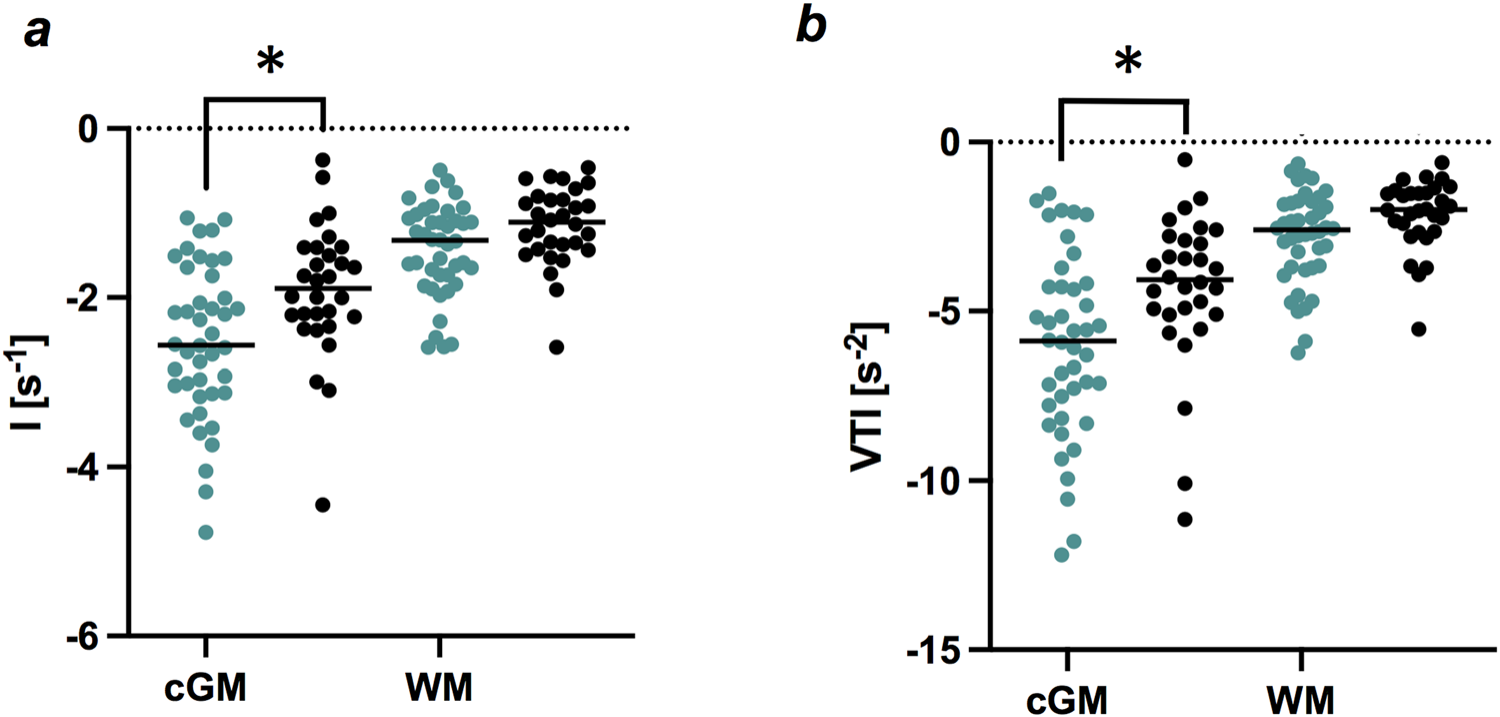
Sex-specific differences in vessel types for grey and white matter, measured with (a) parameter I and (b) vessel type indicator (VTI). Mean values per VOI, for women (green, n = 42) and men (black, n = 30). Line indicates median value. Asterisks indicate FDR-adjusted p-values for comparison between women (n = 42) and men (n = 30), Mann-Whitney-test. *p<0.05. [cGM: cortical grey matter, WM: white matter].

Capillary-bed sensitive parameter CBI did not correlate with age in any of the analyzed VOIs. However, we found significantly higher CBI-values for women in cortical grey matter across all age groups, in both cGM (p = 0.027) and insular cortex (p = 0.027). Moreover, women had lower mean values for vessel-type sensitive parameters I (p = 0.032) and VTI (p = 0.032) in cGM; see Figure 8a and 8b.

In insular cortex, female subjects across all age groups had on average smaller vessels than age-matched men, as measured by VSI (p = 0.024), and a significant increase with age in both vessel-caliber sensitive parameters VSI (r = 0.37, p = 0.02) and CGI (r = 0.34, p = 0.03). No age-related increase was observed for men (p = 0.50 and p = 0.23, respectively). Furthermore, while mean values for I and VTI did not differ between age-matched female and male subjects in insula, only women showed a significant increase in with age (VTI: r = 0.45, p = 0.003; I: r = 0.37, p = 0.02) whereas men did not (p = 0.26 and p = 0.12, respectively).

When comparing microvasculature in hippocampus, women again displayed smaller VSI (p = 0.024); see Table 3.

#### 3.2.2 Basal ganglia and thalamus

In thalamus, female subjects had higher microvessel density Q (p = 0.025) and a trend towards smaller VSI (p = 0.084) compared to age-matched males; see Table 3. No significant differences were observed between sexes for vessel-caliber sensitive parameter CGI in thalamus or in any other analyzed brain area. However, for women, both VSI (r = 0.34, p = 0.03) and CGI (r = 0.39, p = 0.01) significantly increased with age, an effect not observed in age-matched men (VSI: r = 0.25, p = 0.20; CGI: r = 0.34, p = 0.07). In striatum, that is putamen and CN, women had higher microvessel density Q than men; see Table 3. Additionally, we observed smaller VSI (p = 0.024), in female subjects in putamen, but not in CN. Globus pallidus only showed sex-specific differences in microvessel density Q, with higher values for women (p = 0.025).

#### 3.2.3 White matter

In white matter, no significant sex-specific differences were observed after correcting for multiple comparisons. However, there was a trend towards lower, for example, more negative values for parameters I (p = 0.084) and VTI (p = 0.076) in women; see Figure 8a and 8b.

## 4 Discussion

To our knowledge, this is the first study to characterize age- and sex-specific changes in cerebral microvascular architecture across different anatomical regions using vascular architecture mapping. Main findings of our study were i) that microvascular morphology and aging-related remodeling differ between sexes, with differences most pronounced in thalamus, insular cortex, and putamen, ii) that dominant vessel types change with aging, from capillaries to an arteriole-dominated profile, particularly in insula, thalamus, and globus pallidus, iii) that blood flow velocity significantly decreases with age in white, but not in grey matter, and iv) that vessel size is smaller in women and increases with age, while microvessel density is higher in women and decreases with age.

Our results concur with previous studies on aging effects in that we found a decrease in microvessel density, an increase in vessel diameter, with smaller baseline diameter in women, and regional alterations of prevalent vessel type with older age (Bell & Ball, 1981; Berthiaume et al., 2022; Cai et al., 2023; X. Xu et al., 2017).

Even in the absence of cardiovascular risk factors such as diabetes and hypertension (Brown & Thore, 2011; Finger et al., 2022; van Dinther et al., 2022), we found microvessel density to decrease with age, particularly in the basal ganglia, that is CN, putamen and thalamus, regions that are predilection sites for small vessel disease (Caplan, 2015). While microvascular density decreased with healthy aging, women had higher microvessel density compared to age-matched males, in all analyzed grey matter regions but not in white matter. Further, average vascular caliber, as measured with parameters VSI and CGI, increased with age, particularly in CN, thalamus, and insular cortex. While VSI has been shown to be increased in patients with vascular dementia, in both basal ganglia and thalamus (Choi et al., 2020), our results indicate that even in healthy subjects subclinical changes in microvessel caliber occur as part of the normal aging process, matching animal models that show persistent capillary dilatation with age (Bell & Ball, 1981; Berthiaume et al., 2022; Hartmann et al., 2021). Furthermore, computer simulations (Emblem et al., 2013) have suggested that larger values in CGI also reflect reduced capillary recruitment, which occurs with loss of capillary density, as seen with age (Serné et al., 2001). While there was no difference in CGI between sexes, VSI was significantly smaller in our female subjects compared to age-matched males, in line with previous studies (Pabbidi et al., 2018). This matches epidemiological data that incidence of vascular dementia is higher in men than women across all age groups (Ruitenberg et al., 2001). While women had smaller baseline vessel calibers, age-related increases in average vessel calibers were particularly pronounced in women, in both thalamus and CN, a brain area with a particularly high density of steroid-receptors (Honarpisheh & McCullough, 2019). The majority of our female subjects were premenopausal. We therefore hypothesize that these sex-specific differences in vessel caliber and density are partly attributable to sex hormone differences, matching clinical data that earlier menopause is associated with higher risk for ischemic strokes in women (Welten et al., 2021).

While regional cerebral blood volume increased with age in both grey and white matter, women showed a trend towards lower rCBV compared to age-matched males in thalamus and hippocampus, regions that have been found to show sex differences in volume and tissue density (Honarpisheh & McCullough, 2019; Ruigrok et al., 2014). However, these results did not reach statistical significance after correcting for multiple comparisons. Previous studies comparing cerebral blood flow between women and men have yielded contradictory results, either higher (Aanerud et al., 2017) or lower (Barnes, 2017) blood flow in women, with differences most pronounced in premenopausal women. Our results suggest that sex-specific differences in rCBV depend on the brain region that is analyzed, but need further investigation.

On average, all investigated brain regions exhibited a relative dominance of venules and capillary-type vessels in healthy tissue, as indicated by negative values for VTI. While women had more negative values than men, particularly in cGM, healthy aging was associated with an increase in VTI, as seen in insular cortex, GP, and thalamus. Increased age was associated with positive values for VTI, pointing again towards an age-related loss of capillary-type vessels and as a result, relative dominance of fast-flowing arterioles, matching histological studies that show decreased capillary, but increased arteriole density with aging (Bell & Ball, 1981). Age-related modification in microvessel type was particularly pronounced in insular cortex, an area crucial to social cognition, with potential implications for age-related changes in social behavior and interactions (Rogers-Carter et al., 2018; Siracusa et al., 2022). When separating by sex, these changes were only seen in the female subgroup.

An age-related decrease in cerebral blood flow velocity, as measured with VIPS, was only observed in WM, but not in grey matter in both men and women. White matter has been shown to be particularly vulnerable to chronic hypoperfusion, with hypoperfusion-associated white matter damage being observed in both subcortical vascular dementia, and Alzheimer’s Disease, but also normal aging (Chen et al., 2022; Hopkins et al., 2006; Moody et al., 1990; Zhai et al., 2016). Future longitudinal research should explore the predictive value of changes in VIPS with regard to white matter damage and cognitive decline.

In order to analyze physiological age-related changes, we included only vascular healthy subjects, spanning an age range of five decades. However, the majority of our female subjects was under 50 years of age, and therefore likely premenopausal, where hormonal differences in vascular properties, such as blood flow or vascular tone, have been shown to be most pronounced (Kastrup et al., 1998; Pabbidi et al., 2018). The described sex-specific differences may therefore be more pronounced than in the general population. Furthermore, the vast majority of our cohort were white Caucasians. Future research should take into account race-related differences in microvasculature changes as well.

Lastly, we analyzed only contralateral hemispheres from clinically stable patients with unifocal WHO grade 1-2 lesions, who had not received any radio- or chemotherapy prior to the MR scan. Still, one limitation of our study remains that data were collected from non-compromised brain hemispheres of patients with contralateral low-grade brain tumors. While it was hypothesized that a small fraction, approximately 10%, of a subtype of these brain tumors, that is, diffuse low-grade glioma, corresponding to 3 of 72 patients of our study, may be prone to tumor cell migration into the opposing brain hemisphere (Fukuya et al., 2019), the examined hemispheres did not show any radiological signs of tumor cell infiltration. In addition, no diffuse low-grade glioma patient had a new contralateral lesion in follow-up exams. Even if macroscopically hidden lesions were present, considering that typical standard deviations in our cohort were within 30% of mean values, changes in 4% of mean value constituting parameters will not significantly change mean values or standard deviations within this range, therefore having no effect on our results and conclusions.

In summary, we found that age-related changes in microvascular arrangements differ between sexes. While microvascular arrangements in women overall resembled younger microvascular profiles, aging effects, particularly in thalamus and insular cortex, were more pronounced in women. These effects should be taken into account when conducting further VAM studies, and might serve as a starting point in sex-specific diagnostics and prevention of cerebrovascular disease.

## Data Availability

Data and Code are available upon reasonable request from a qualified investigator with the need of a formal data transfer agreement, due to ethical and legal restrictions involving potentially identifying patient information, imposed by the ethics committee of the medical faculty of Heidelberg University (Alte Glockengießerei 11/1, 69115 Heidelberg, Germany). Data requests may be sent to the Department of Neurology, Heidelberg University Hospital (Im Neuenheimer Feld 400, 69120 Heidelberg, Germany) or directly to the corresponding author.
